# Nanomechanical effects of light unveil photons momentum in medium

**DOI:** 10.1038/srep42554

**Published:** 2017-02-15

**Authors:** Gopal Verma, Komal Chaudhary, Kamal P. Singh

**Affiliations:** 1 Department of Physical Sciences, Indian Institute of Science Education and Research Mohali, Sector-81, Manauli 140306, India

## Abstract

Precision measurement on momentum transfer between light and fluid interface has many implications including resolving the intriguing nature of photons momentum in a medium. For example, the existence of Abraham pressure of light under specific experimental configuration and the predictions of Chau-Amperian formalism of optical momentum for TE and TM polarizations remain untested. Here, we quantitatively and cleanly measure nanomehanical dynamics of water surface excited by radiation pressure of a laser beam. We systematically scanned wide range of experimental parameters including long exposure times, angle of incidence, spot size and laser polarization, and used two independent pump-probe techniques to validate a nano- bump on the water surface under all the tested conditions, in quantitative agreement with the Minkowski’s momentum of light. With careful experiments, we demonstrate advantages and limitations of nanometer resolved optical probing techniques and narrow down actual manifestation of optical momentum in a medium.

The nanoscopic surface deformation of a transparent dielectric fluid illuminated by light results from transfer of momentum from photons to the interface. More than a century ago, Minkowski predicted that photons would gain momentum *P*_*M*_ = *nP*_0_ compared to its value in vacuum *P*_0_, upon entering a medium of refractive index *n*^1^. However, Abraham predicted a loss in its momentum cite ref. 2, *P*_*A*_ = *P*_0_/*n*. The conservation of momentum at the air-water interface implies that light would cause an outward bulge or a dimple, if Minkowski or Abraham momentum is assumed to be correct, respectively. These conflicting predictions resulted in a dilemma over which many theoretical and experimental contributions were reported^3^,^4^,^5^. Furthermore, it was proposed that these momenta are not intrinsic nature of light but emerge due to their interaction with the medium^3^. Meanwhile the extraordinary momentum in various light-fields have been demonstrated^6^,^7^. Theoretically, Abraham and Minkowski momenta were identified as kinetic and canonical momenta, respectively^8^,^9^,^10^. However, it was left for experiments to decide which one would manifest for a given situation. A clean and quantitative validation of optical momentum is non-trivial because of weak signals, typically nanometric with Watt level laser beams on air-water interface.

Ashkin and Dziedzic made the first observation of a transient bulge on free water surface using tightly focused *ns* pulses with kW peak power^11^. Large stationary bulge was reported using critical fluid-fluid interfaces having ~*μN/m* surface tension, besides these systems also exhibited interesting nonlinear optofluidic responses^12^,^13^. The optical probing techniques with nanometric precision have been applied to probe laser-induced surface deformations^14^,^15^. Many experiments were performed with laser exposure on *ms* or faster time scales and reported a nano-bulge^12^,^13^,^14^,^15^. Recently, the first claim of the Abraham pressure of light on water, i.e., a dimple, was reported for about one second exposure of a collimated laser beam and for large water depth^4^. The long exposure times was argued to generate a characteristic flow pattern in water thus causing a dimple. A subsequent experiment did not observe the dimple, but the exposure time was not same as in the original experiment and no explanation for the absence of dimple was given^5^. These previous conflicting experimental results continued the momentum debate.

Besides, the Abraham and Minkowski momenta^3^,^8^,^9^,^16^,^17^, three other momentum densities known as Einstein-Laub, Amperian and Chu have also been proposed. The experimental resolution of their existence has remained inconclusive^4^,^5^. Most previous experiments with quasi-normally incident laser^5^,^11^,^12^,^15^ could not resolve weak polarization dependence of laser-driven deformation. A quantitative observation of polarization dependent radiation pressure effects (dip/bump) can further narrow down various energy-momentum formulations. For example, Amperian and Chu formulation postulate, an upward bulge for TE polarization, which is smaller in magnitude compared to other formalisms, and a dip for TM polarization^18^. It is therefore critical to devise rigorous and clean experiments under different configurations with independent cross-checks using multiple probing techniques to rationalize the observation.

Here, we investigated the nano-mechanical effects of photons momentum at the air-water interface using pump-probe optical techniques in various geometries. We systematically scanned experimental parameters like exposure time, polarization, spot-size, water height, incidence angle and measured the water surface deformation with nano-meter precision using two optical pump-probe setups. We cleanly demonstrated an evidence of Minkowski’s pressure of light at air-water interface under all tested conditions. In addition, we show why the pump-probe techniques are advantageous over the single-beam methods employed in some previous works.

## Results

### Nanometric optical probes

Two independent pump-probe setups were used to quantify nanometric dynamics of the water surface illuminated by a pump beam. In both the cases, the deformation in the medium was induced by focusing a linearly polarized Gaussian (pump) laser beam on the air-water interface at an angle of incidence *θ*_*i*_. The water height was varied up to 10 *cm* in a transparent cubic or cylindrical pot. Experiments were also performed, in a different configuration, with a water drop resting on the horizontal surface of a glass prism.

**Figure 1:**
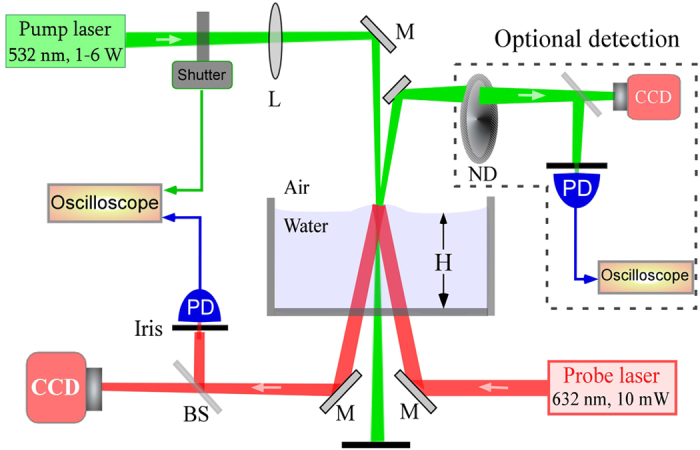
Schematics of the experimental set-up. Interface deformation was detected using a probe and the optional detection. L: lens, BS: beam splitter, PD: photodiode, ND: neutral density filter. The probe beam transmitted above the water surface is not shown.

The surface deformation was optically probed, first using a beam-profile based technique (Fig. 1), which has been previously employed by many authors^15^,^19^,^20^. A low-power collimated He-Ne laser (*λ *= 632 *nm*, 10 *mW*) was made to fall on the water surface from below and the full beam profile of its partial reflection (~2%) from the air-water interface was captured on a CCD camera. The spatial overlap between the pump and the probe beams on the water surface was experimentally optimized to produce the maximum deformation signal keeping other parameters fixed. In addition, the central intensity in the probe beam *I*(*t*), was measured along with the corresponding shutter signal. If the water surface makes a bump (dip), the probe beam will be focused that would reduce (increase) its spot size on the screen. The bump (dip) would therefore produce higher (lower) intensity on the photodiode. Using the Gaussian beam propagation formula as given in Eq. (1), quantitative information of the pump induced deformation was obtained (see Supplementary material)^4^. An optional detection of the beam profile and the central intensity of the pump was also performed.

In the second configuration, the He-Ne laser was used to produce high-contrast Newton-ring like fringes from the sessile water drop on a glass prism as discussed later. We call this setup a liquid drop interferometer (LDI). The LDI technique was previously shown to offers sub-5 *nm* precision in measuring water surface displacement in a self-calibrating way^14^,^21^. The key advantage with this technique is that it cleanly measured the direction and magnitude of the nano-metric deformation (see supplementary material for more details). Since, LDI also simultaneously captured evaporation induced reduction in drop thickness with nanometric precision, it allowed us to cleanly validate optical momentum effect against any thermal effects by the pump beam. Its high sensitivity was used to further isolate weak polarization dependence of the radiation pressure effects in oblique incidence.

### Nanometric bulge

**Figure 2:**
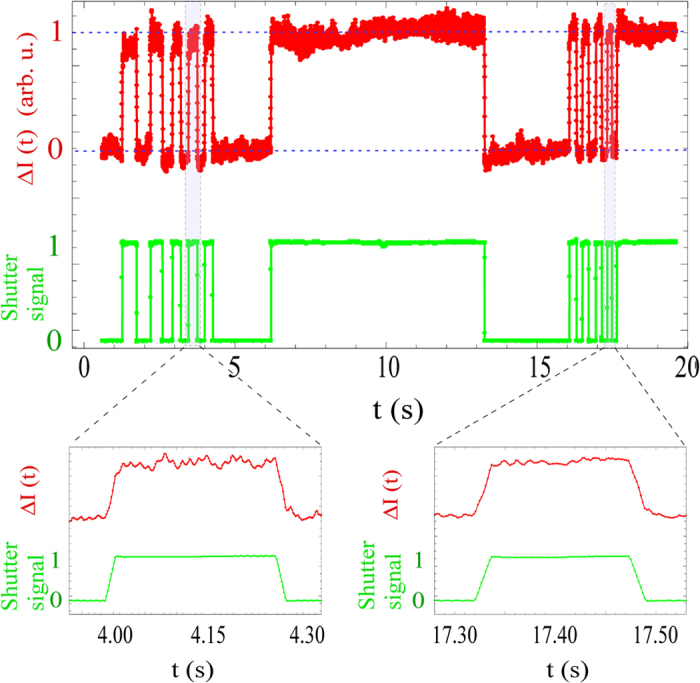
Probe intensity Δ*I*(*t*) = *I*(*t*) − *I*_0_ for multiple pump exposure times along with corresponding shutter signal. Experimental parameters: *P* = 2.5 *W, w*_0_ = 100 *μm*. Zoom of the two events is shown below.

The intensity profile of the probe beam after its reflection from the flat water surface (without the pump) served as a reference and its central intensity *I*_0_ corresponded to undeformed water surface. Figure 2 shows temporal evolution of Δ*I*(*t*) = *I*(*t*) − *I*_0_ along with the corresponding shutter signal. Multiple flashes of the pump beam with ~10 *ms* to ~10 *s* exposure times were recorded. In all the cases, the probe intensity *I*(*t*) increased, followed the pump power then saturated and returned to the original level after it was switched off. The observed focusing of the probe beam indicated a bump on the water surface. Experiments were performed by varying the water height *H* up to 10 *cm* and no dimple was ever seen. Our experiments show that the bump observed in previous experiments on *ms* exposure^14^,^15^ remains valid even for long exposure times. But, no signature of Abraham pressure, i.e., a dimple on the water surface, was observed in our experiments unlike what was claimed in ref. 4.

**Figure 3:**
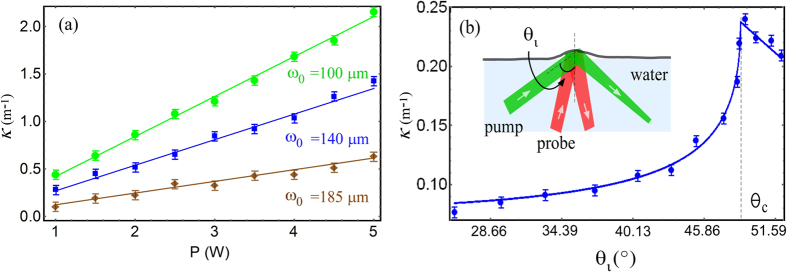
(**a**) Curvature *κ* versus input pump power for three beam waists *ω*_0_. (**b**) *κ* versus incidence angle when pump enters water from below, as shown in the inset. Solid line is a theoretical fit using equation described in text. The error bars indicate standard deviation of experimental noise floor.

Quantitative measurements of the radius of curvature *κ* = 1/*R* of the laser-induced bump is shown in Fig. 3(a). The radius of curvature *R* was calculated using the Gaussian beam propagation for the probe,


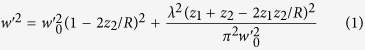


where *z*_1_ and *z*_2_ are distances from the laser to the interface and from the interface to the CCD camera, respectively (see Supplementary material for details). *w′*_0_ and *w*′ are respectively the 1/*e*^2^ beam radii of input beam and on the CCD.

The experimental data were fitted with the theoretical curvature at the central position of the bump, 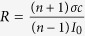, where 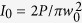 is the maximum intensity, *c* is the speed of light^4^. *σ* and *n* denote surface tension and refractive index of water, respectively. The experimental data for *κ* exhibited a linear dependence on the input pump power for three different values of 1/*e*^2^ pump beam radius *ω*_0_ of 100 *μm*, 140 *μm*, and 185 *μm* on the water surface (with corresponding Rayleigh ranges 12 *cm*, 23 *cm*, 40 *cm*, respectively). Our spot sizes were close to the ones used in^4^ which reported 1/*e*^4^ beam radius of 264 *μm* on the water surface. A good quantitative agreement with the theoretical fit using the Minkowski momenta for photons was evident.

### Enhancement near total internal reflection

Few experiments were performed previously to rigorously test optical momentum at an oblique incidence near TIR. We measured the deformation of water surface by varying the angle of incidence of the pump *θ*_*i*_ irradiated from below the water surface (see inset of the Fig. 3(b)). Figure 3(b) shows our measurements of *κ* versus *θ*_*i*_ for *P* = 2.8 *W* pump power. Our data with the maximum deformation height at the center of the bump for oblique incidence angle was given by^14^


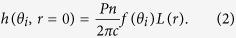


where the spatial dependence is governed by the integral, 
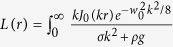
 *dk* and *J*_0_ is zeroth-order Bessel function. *f*(*θ*_*i*_) defines angular dependence of the reflection coefficients of the water surface which depends on the input polarization (Supplementary information). For the pump beam illuminating the water surface from below, a unique bump was observed for all *θ*_*i*_ and input polarizations. The magnitude of the bump smoothly increased with incidence angle and became about three times larger near TIR when compared to the quasi-normal incidence. The enhancement in *κ* near the critical angle was due to increase in the reflection coefficient of the interface thus enhancing the momentum transfer to the interface. Our data did not agree with previous claim of a dimple with low-power laser^22^,^23^,^24^,^25^,^26^, but agreed with the pump-probe measurement of a long-range bump on the sessile water drop^14^.

### Importance of probe beam

We now turn our attention to the optional detection scheme in Fig. 1 where the partially reflected pump beam was used to probe the interface deformation.

Such single-beam method was previously used in many experiments^4^,^11^,^22^. It is necessary to understand their capability and limitations to probe weak curvature effects in order to rationalize any experimental observation. In our set-up, for *P* = 1–5 *W* pump power, the partial reflection from the water surface (~2%) was much higher compared to the probe beam and this saturated the CCD camera or the photodiode. We had to therefore attenuate the reflected pump beam power considerably using a standard ND filter (Thorlabs, OD-4) to be able to make any measurements.

**Figure 4:**
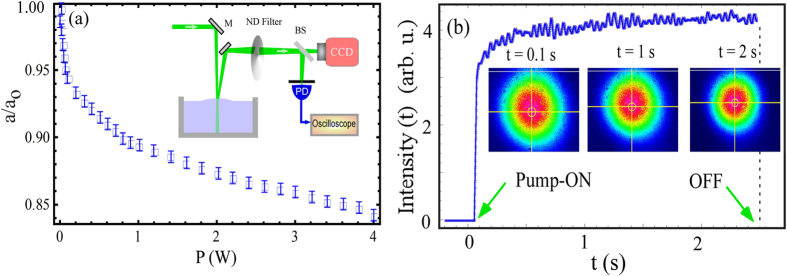
Beam profile distortion by absorptive filter in optional measurement of Fig. 1. (**a**) *a/a*_0_ denote ratio of beam diameters measured on the screen *a* and its value for low-power *a*_0_. Note that diverging reflection of the pump appears focused after ND filter. (**b**) Photodiode signal when the pump beam is switched on and off. The beam profiles for *t* = 0, *t* = 1 *s* and *t* = 2 *s* show a focusing effect. The slight oscillations in the PD signal is due to fluctuations on the water surface.

One would expect that the bump on the water surface would cause a defocussing of the reflected pump beam on the screen. However, we observed that the beam diameter after the ND filter was reduced suggesting the focusing effect. As shown in Fig. 4(a), the ratio *a/a*_0_ of beam diameters on the screen *a* to the initial beam waist *a*_0_ continuously decreased with the increase in input power. Surprisingly, the observed self-focusing in the pump beam was caused by the thermal deformation in the ND filter. We tested many different filters and observed similar focusing effect in the pump beam for different pump powers and input beam diameters. The self-focusing by the ND filter competed against the weak defocussing due to the bump on the water surface. For higher pump powers, larger optofluidic deformation increased the spot size on the filter which reduced the slope of *a/a*_0_. This instrumental effect should not be confused with the radiation-pressure effect on the fluid interface, although it appeared linear for some power ranges.

The time-scale associated with the self-focusing effect in the filter was measured in Fig. 4(b). After switching on the pump beam, its profile focused gradually and stabilized after ~2 *s*. Such single beam detection allow optofluidic measurement over a limited time interval only when the pump beam is present. The lack of flexibility in this technique does not always allow independent experimental cross checks against possible artifacts due to intense pump beam. The time-scales around one second was previously observed in a similar experiment and was associated with the bulk flow of water, however, the flow was not measured^4^. Our demonstration clearly suggested that the single-beam setup should be implemented carefully because these could obscure the weak signal from the optofluidic curvatures. It is therefore critical to employ an independent probe beam.

### Polarization dependence of nanobulge

**Figure 5:**
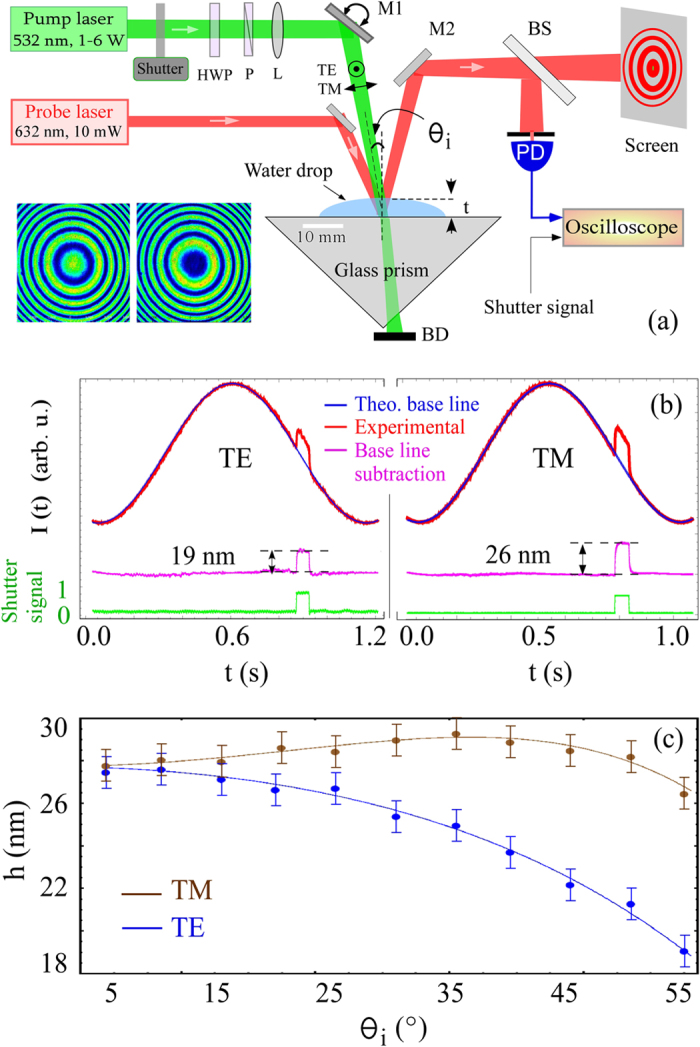
(**a**) Schematic of pump-probe type liquid drop interferometer setup. The pump beam enters the water drop from above. The high-contrast circular fringes in the probe obtained from a water drop are recorded with a beam profiler as shown in the lower left corner. HWP: half wave plate, P: polarizer, L: lens, BS: beam splitter, PD: photodiode, BD; beam dump. (**b**) PD signals for water height for a pump exposure for TE and TM polarizations. Fitting the data with a cosine curve, excluding the pump exposure, was used as evaporation base-line. Base-line subtracted signals are shown along with shutter signal. Here, *θ*_*i*_ = 55°, *P* = 2.8 *W, ω* = 50 *μm*. (**c**) Deformation for TE and TM polarizations versus *θ*_*i*_. Solid lines are theoretical fits using Eq. (2).

To test if change in linear polarization (TE/TM) can induce any dimple on air-water interface, as predicted by Chau-Amperian formalism^18^, we employed the LDI pump-probe setup (Fig. 5(a)). This technique will independently cross-check the optical momentum effects described previously. The LDI offers many advantages^14^,^21^. It is self-calibrating with sub-5 *nm* precision. In addition, we also measured the nanomtric reduction in the height of the water due to evaporation, the absence of thermal effects in our measurements is also established.

Figure 5(b) shows our measurement of the deformation height, obtained after baseline subtraction, of a sessile water drop along with corresponding shutter signal controlling pump on-off. We clearly observed an outward bump in response to the pump laser. The height of the bump was higher by about 7 *nm* for TM polarization when compared to TE for oblique angle (Fig. 5(c)). These measurements were in quantitative agreement with the Minkowski momentum, assuming a balance of forces as described above. The prediction of Chau-Amperian formalism for a dimple with TM polarization was not validated.

## Discussion

All the previous experiments with pump-probe type techniques reported an outward bump on the air-fluid interface. So far the bump was observed for a wide range of experimental parameters (exposure time, polarization, incidence angle, spot size etc). The observation of bump with nanometric precision on the air-water interface was also in agreement with large deformation observed on the critical fluid-fluid interface^12^. Based on so many reliable experiments on fluid systems, the Minkowski momentum uniquely determines the fluid deformation for both pulsed and cw laser excitation for all range of parameters tested thus far. However, care must be taken to correctly use the single-beam method^4^,^11^,^22^ because the intense laser beams might produce instrumental effects that could mask the true signal. Furthermore, no independent cross checks are generally possible which is necessary to cleanly validate such tiny effects^4^,^22^. These limitations of the experimental technique could be one of the reasons for conflicting experimental claims in the literature. In conclusion, our quantitative and careful pump-probe experiments demonstrated a universal presence of Minkowski momenta on the air-water interface. We systematically scanned various relevant experimental parameters such as pump exposure time, spot size, water height, and angle of incidence and also used independent cross-checks to validate our results. Under all the tested conditions, an outward nano-bulge on the water surface was observed, in quantitative agreement with the Minkowski’s momentum, therefore, Abraham momentum was absent. The demonstrated experimental techniques may find applications in probing many optofluidic phenomena.

## Methods

The experiment was performed with water (Millipore) in cubic pot (10 *cm *× 10 *cm *× 10 *cm*) or cylindrical pot of diameter 10 *cm*. Experiments with sessile water drop were performed for drop of radius around 10 *mm* placed on a horizontal face on an equilateral glass prism of refractive index *n*_*g*_ = 1.55. The contact angle of the drop was near 40° and its central thickness was ~1 *mm*. The water temperature was *T* = 300 ± 0.1° *K* with ambient humidity ~55%. The setup was assembled on an optical table (Thorlabs) floating on air.

The probe laser was a linearly polarized, 10 *mW* He-Ne laser (Mels-Griot) at *λ *= 632 *nm* with a collimated full waist of 1000 *μm*. The pump laser (Coherent) was a *λ *= 532 *nm* green cw laser with 5 *W* maximum power working in *TEM*_00_ mode. The laser power was varied by a 10 *cm* diameter circular ND filter wheel (Thorlabs, OD-4). The laser was focused on air-water interface with a biconvex anti-reflection coated lenses. The 1/*e*^2^ beam diameters for pump and probe were measured by a beam profiler (Thorlabs, BP106-VIS). The laser power was measured with a power meter (Thorlabs, Model PM100D).

The incidence angle *θ*_*i*_ of the pump was varied by rotating a Ag-mirror with motorized rotation stage (Thorlabs). A programmable 12 *mm* clear aperture shutter (Thorlabs) was used to switch on/off the pump beam. The spatial overlap between the pump and probe on the air-water interface was experimentally optimized for generating maximum deformation. A fast photodiode (1 ns rise time, Thorlabs, DET10A) and a 2.5 *Gs* oscilloscope measured the probe laser intensity.

## Additional Information

**How to cite this article:** Verma, G. *et al*. Nanomechanical effects of light unveil photons momentum in medium. *Sci. Rep.* **7**, 42554; doi: 10.1038/srep42554 (2017).

**Publisher's note:** Springer Nature remains neutral with regard to jurisdictional claims in published maps and institutional affiliations.

## Supplementary Material

Supplementary Information
