# Supplementary material for: Nanomechanical effects of light unveil photons momentum in medium
Source: Sci Rep. 2017 Feb 15;7:42554. doi: 10.1038/srep42554 (PMC5309822; doi:10.1038/srep42554)
Supplement: Supplementary Information [file srep42554-s1.pdf]

Supplementary Information

Nanomechanical effects of light unveils photons momentum in medium

Gopal Verma,[1] Komal Chaudhary,[1] and Kamal P. Singh[1]

[1] *Department of Physical Sciences, Indian Institute of Science Education and Research Mohali, Sector-81, Manauli 140306, India.*

1. Experimental determination of interface curvature using Gaussian beam propagation

We calculated the radius of curvature (R) of deformed AW interface using the Gaussian beam propagation formula

$$w'^2 = w_0'^2(1 - 2z_2/R)^2 + \frac{\lambda^2(z_1 + z_2 - 2z_1z_2/R)^2}{\pi^2 w'^2} \quad (1)$$

where z_1 is distances from laser source to AW interface and z_2 from AW interface to CCD camera. Putting the value of z_1 z_2, w' and $w_0'^2$, we have calculated the value of R

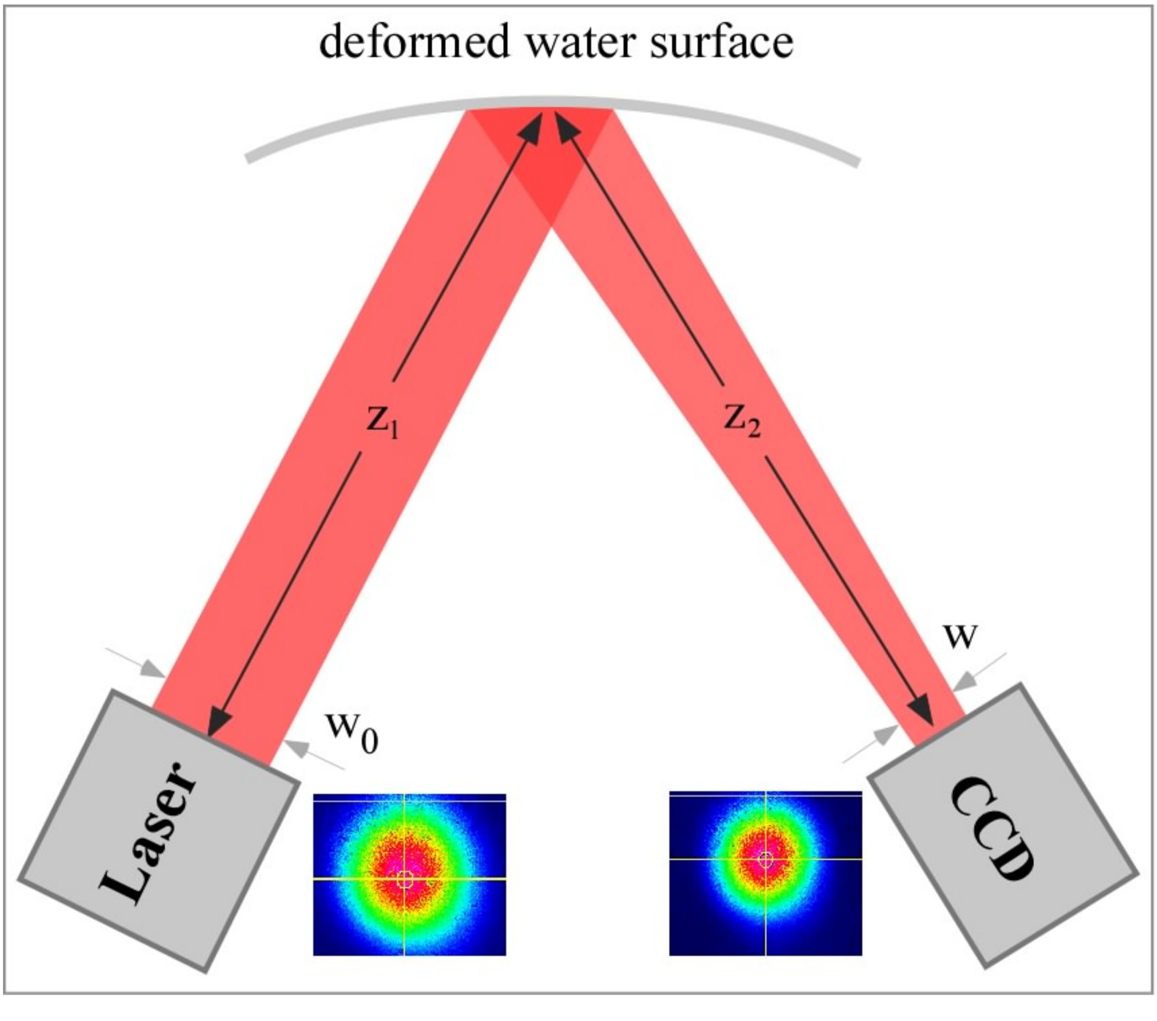

FIG. 1: Schematics of Gaussian beam reflection from the curved air-water interface.

2. Precision and calibration of Liquid drop interferometer set-up

The phase difference between the two reflections for a fluid drop of thickness d and refractive index n_l is given

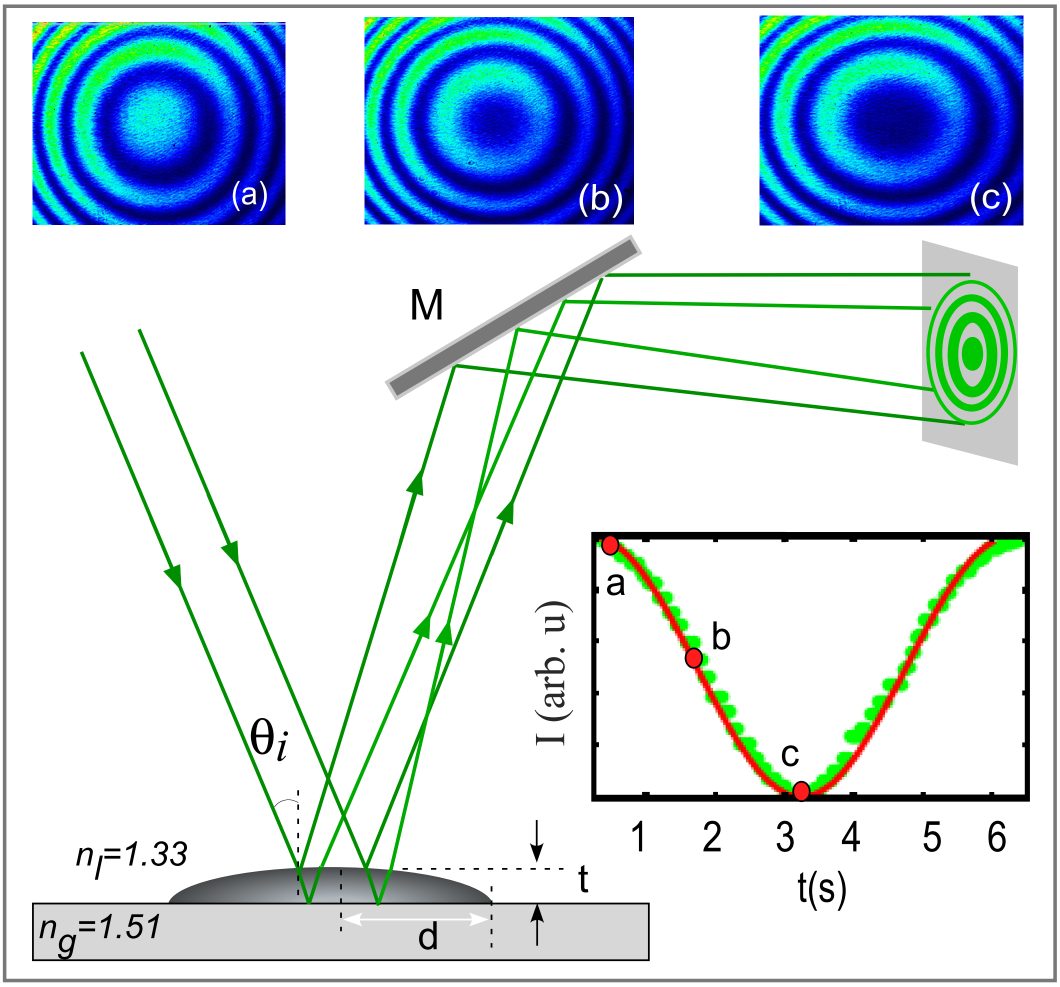

FIG. 2: Schematic experimental set-up of Liquid Drop Interferometer (LDI)

by, $\Delta\phi(t) = (2\pi n_l/\lambda)(2d(t)/cos\theta_r)$, where θ_r is determined by the Snell's law, $n_l = \sin\theta_i/sin\theta_r$. This dynamical interference pattern was used to extract time-resolved height variation. Note that this technique is self-referencing for nanoscale measurements. One fringe collapse, i.e., central maximum to the next minimum of $I(t)$, as shown in Fig. 2(a)-(c), corresponded to thickness change of $\lambda\cos\theta_r/4n_l \approx 117\ nm$ in our experimental conditions! Interestingly, by fitting experimental intensity variation to the theoretical one we obtained $< 5\ nm$.

3. Deformation height and curvature

Direction and magnitude of bulge supports Minkowski's form of momentum transfer to the AW interface. To generate a stationary deformation of AW interface the radiation pressure is balanced by both buoyancy and Laplace pressure. It leads to,

$$\rho g h(\theta_i, r) - \sigma\kappa(r) = \Pi(\theta_i, r). \tag{2}$$

Where ρ is the density of water, σ is the surface tension, and $\kappa(r)$ is curvature of the interface. Following the Minkowski's formalism, radiation pressure of a Gaussian laser beam obliquely incident at AW interface from water is given by,

$$\Pi(\theta_i, r) = \frac{n_i I(r)}{c} f(\theta_i). \tag{3}$$

Here $f(\theta_i) = \cos^2\theta_i(1 + R - \dfrac{\tan\theta_i}{\tan\theta_t}T)$ θ_i and θ_t denote angles of incidence and transmission, respectively and c is the

speed of light. The polarization dependent reflection and transmission coefficients are given by

$$R_{TE} = \left(\frac{n_i cos\theta_i - n_t cos\theta_t}{n_i cos\theta_i + n_t cos\theta_t} \right)^2 \tag{4}$$

$$R_{TM} = \left(\frac{n_t cos\theta_i - n_i cos\theta_t}{n_t cos\theta_i + n_i cos\theta_t} \right)^2 . \tag{5}$$

Magnitude of AW interface deformation height was determined by

$$h(\theta_i, r = 0) = \frac{P}{2\pi} \frac{n_i}{c} f(\theta_i) L(r). \tag{6}$$

Where the spatial dependence is governed by the integral, $L(r) = \int_0^\infty \frac{k J_0(kr) e^{-w_0^2 k^2/8}}{\sigma\ k^2 + \rho\ g} dk$ and J_0 is zeroth-order Bessel function. For the maximum 5 W pump power and normal incident ($\theta_i = 0$) the bump height was $\simeq$ 25 nm. Radius of curvature for general angle of incident is given by $h''(\theta_i, r = 0)$. For a water of refractive index n , surface tension σ at normal incident radius of curvature is given by $R = \frac{(n+1)\sigma c}{(n-1) I_0}$, where $I_0 = 2P/\pi w_0^2$ is the maximum intensity at AW interface with laser power P.
